# Effects of underestimating the kinematics of trunk rotation on simultaneous reaching movements: predictions of a biomechanical model

**DOI:** 10.1186/1743-0003-10-54

**Published:** 2013-06-12

**Authors:** Martin Simoneau, Étienne Guillaud, Jean Blouin

**Affiliations:** 1 Faculté de Médecine, Département de kinésiologie, Université Laval, Québec, Canada; 2 Vieillissement, Centre de recherche FRSQ du CHU de Québec, Québec, Canada; 3 INCIA, CNRS UMR, Université de Bordeaux, Bordeaux, 5287 France; 4 Laboratory of Neuroscience of Cognition and Cognition, CNRS and Aix-Marseille University, Marseille, France

**Keywords:** Biomechanical model, Torso rotation underestimation, Reaching accuracy

## Abstract

**Background:**

Rotation of the torso while reaching produces torques (e.g., Coriolis torque) that deviate the arm from its planned trajectory. To ensure an accurate reaching movement, the brain may take these perturbing torques into account during movement planning or, alternatively, it may correct hand trajectory during movement execution. Irrespective of the process selected, it is expected that an underestimation of trunk rotation would likely induce inaccurate shoulder and elbow torques, resulting in hand deviation. Nonetheless, it is still undetermined to what extent a small error in the perception of trunk rotations, translating into an inappropriate selection of motor commands, would affect reaching accuracy.

**Methods:**

To investigate, we adapted a biomechanical model (J Neurophysiol 89: 276-289, 2003) to predict the consequences of underestimating trunk rotations on right hand reaching movements performed during either clockwise or counter clockwise torso rotations.

**Results:**

The results revealed that regardless of the degree to which the torso rotation was underestimated, the amplitude of hand deviation was much larger for counter clockwise rotations than for clockwise rotations. This was attributed to the fact that the Coriolis and centripetal joint torques were acting in the same direction during counter clockwise rotation yet in opposite directions during clockwise rotations, effectively cancelling each other out.

**Conclusions:**

These findings suggest that in order to anticipate and compensate for the interaction torques generated during torso rotation while reaching, the brain must have an accurate prediction of torso rotation kinematics. The present study proposes that when designing upper limb prostheses controllers, adding a sensor to monitor trunk kinematics may improve prostheses control and performance.

## Background

Arm movements are among the most frequent and important actions in the human voluntary motor repertoire. These complex movements enable us to feed and look after ourselves as well as others, build safe shelter and perform other life-sustaining activities. Reaching is a complex motor action as shoulder and elbow joint torques arise not only from muscles acting at both joints, but also from interactions due to the movement of other limbs. For instance, extending the elbow while flexing the shoulder generates an interaction torque at the shoulder joint. These torques depend, in a nonlinear fashion, on the motion of adjacent joints. Both behavioral and simulation studies indicate that a failure of the motor commands to account for the interaction torques results in severe disturbances of the movement trajectory [1-3]. Understanding the biomechanical and sensorimotor control processes involved in reaching may help clinicians identify the location of deficits occurring in individuals with neurological disorders.

Arm movements often occur simultaneously with trunk rotations, such as when approaching an object out of reach on our side. Moving the trunk can also be used as a strategy to move the arm in space when arm movements are impaired as a result of brain damage [4]. Importantly, trunk rotation during reaching produces Coriolis torques that push the arm perpendicularly to the hand-velocity vector and in the opposite direction of the rotation. Without taking into account these torques, it would be impossible for the brain to command smooth and accurate arm movements. These movements may include those that accompany our own displacements or more specifically, those that we admire in sports and dance. The inertial Coriolis force is dependent on the cross product of the linear velocity of the arm and the angular velocity of torso rotation. Several studies have shown that when body rotations cannot be detected accurately, as during sustained passive body rotation at a constant velocity [5], the trajectories and endpoints of reaching movements are first deviated in the direction of the Coriolis force applied on the arm [6-9]. After a few movements produced under such conditions, the hand trajectory straightens, thereby increasing endpoint accuracy e.g., [8]. It is believed that this improvement is a result of motor adaptations to Coriolis perturbations. Furthermore, Pigeon et al. [5] showed that when we reach for an object while simultaneously rotating the torso, despite the potential for trunk motion to perturb arm movement, the reach is still accurate. This observation holds true even in the absence of visual feedback from the hand. The authors demonstrated that, under these circumstances, one does not minimize the Coriolis torques incumbent on trunk rotation by sequencing the arm and trunk motions into a turn followed by a reach. Rather, when reaching for an eccentric object, we generally move both the arm and trunk simultaneously. In building an inverse dynamic model of unrestrained reaching movements, Bortolami and colleagues [10,11] showed that the Coriolis torques at the shoulder joint could be nearly six times larger with torso rotation compared to without. One way to maintain movement accuracy while simultaneously reaching and rotating the trunk would be to correct the deviations of hand trajectory that result from the additional Coriolis torques evoked from the rotation. However, experimental studies have suggested that the brain predicts the consequences of Coriolis torques either prior to or during trunk rotations [5,12]. In either case, a reliable estimate of head-trunk kinematics appears to be necessary in order to assess the mechanical consequences for the reaching arm.

The vestibular system provides feedback regarding the linear and angular motion of the head over a wide range of velocities and frequencies relative to the outside world [13]. The integration of this information with proprioceptive input from the neck muscles provides the brain with information on trunk motion [14,15]. There is evidence that the brain may use information regarding trunk movements to predict the perturbing effects of torso rotation on reaching movements [16,17]. However, there are several instances where vestibular perception of body rotation is impaired. For instance, perception of motion may deteriorate with age [18], various diseases (e.g., midline cerebellar lesions [19], vestibular neuronitis [20], idiopathic scoliosis [21]) and body rotation at a constant velocity [6-8]. In such situations, where the detection of trunk kinematics is compromised, reaching movements should be less accurate. However, it is unknown to what extent reaching accuracy deteriorates as a result of errors in the perception of trunk rotation. On the other hand, because the direction of shoulder and elbow torques depends upon the direction of torso rotation, it is possible that the relationship between underestimating torso rotation and reaching error is different for clockwise and counter clockwise rotations. To our knowledge, there is no straightforward procedure to investigate these issues in human or animal subjects. One major difficulty involves assessing the subject’s’ perception of their rotation while they are simultaneously engaged in a reaching task. Reaching errors may also result from errors in movement planning or in controlling arm movements without visual feedback [22-24].Therefore, the trajectory deviations produced by humans during torso rotation would not provide a direct estimate of the effects of miscalculating trunk rotations on reaching movements. In this context, the use of a biomechanical model emerges as an effective means to determine the consequences of underestimating torso rotation on simultaneous reaching movements. Here, we adapted the biomechanical model of right hand reaching movements proposed by Pigeon et al. [5] to address the following questions: i) to what extend is the brain required to alter reaching motor commands to ensure accurate hand trajectory despite torso rotation?, ii) what is the effect of underestimating torso rotation on right hand reaching accuracy? and, iii) does underestimating counter- versus clockwise rotations have the same influence on reaching accuracy?

## Methods

### Biomechanical model

**Figure 1:**
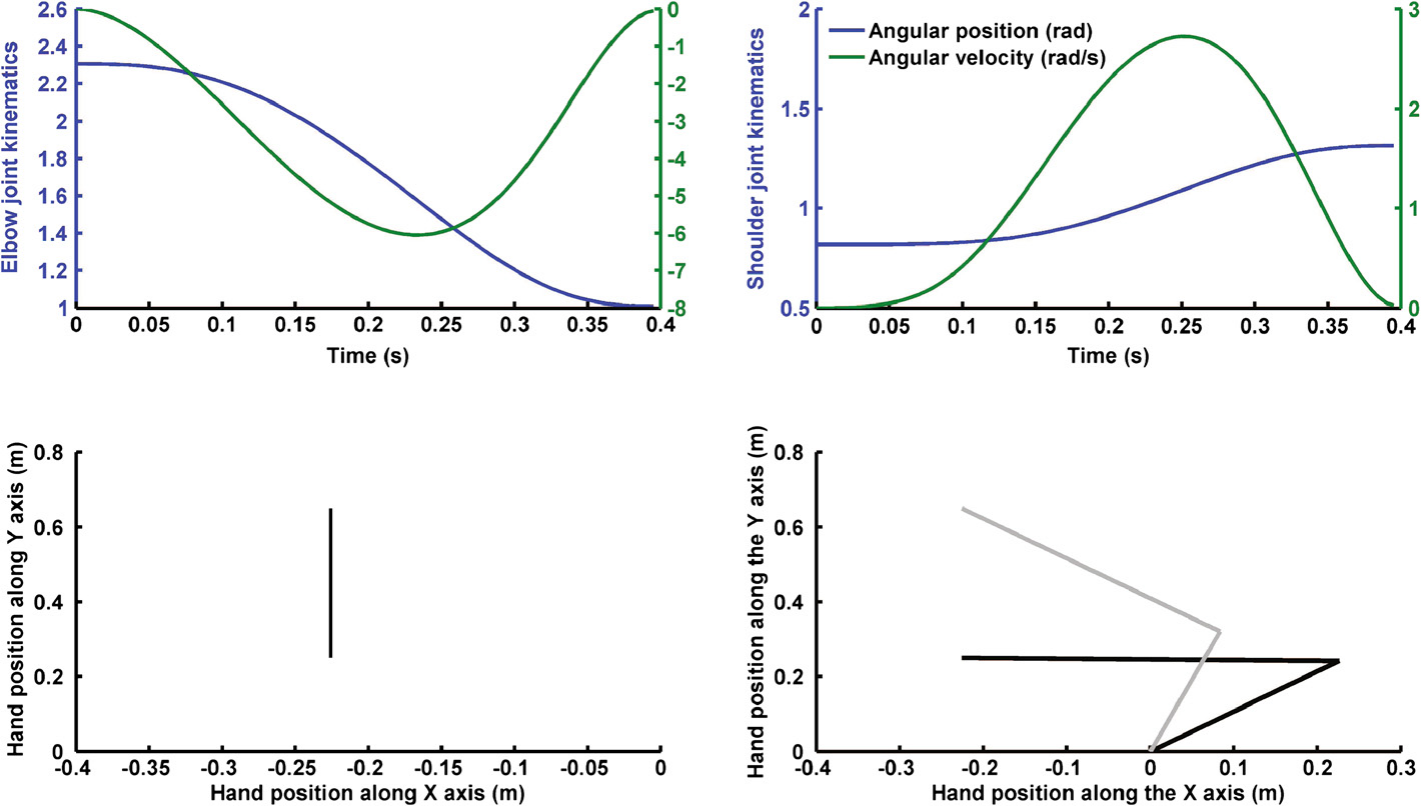
**Upper left and right panels) Elbow and shoulder joint angular position (blue lines) and velocity (green lines) during accurate reaching movements performed in the absence of torso rotation.** Lower left panel) Hand trajectory during the simulated reaching movement. Lower right panel) Initial (black lines) and final (grey lines) arm positions. The shoulder joint is located at the origin of the Cartesian reference frame. The initial hand position is located at (−0.228 0.25) m and the final hand position is located at (−0.2258, 0.65) m.

The reaching arm was considered as two interconnected rigid links (upper arm and forearm) with frictionless joints at the shoulder and elbow. Movement of the right hand was executed in the horizontal plane and followed a minimum jerk trajectory [25]. Hogan [26] showed that the trajectory that minimizes this cost function (i.e., minimum jerk) was a fifth order polynomial equation:

(1) $$\begin{array}{c} x\left(t\right)=x_{0}+(x_{f}-x_{0})\times [10\times {\left(\frac{t}{T}\right)}^{3}-15\times {\left(\frac{t}{T}\right)}^{4}+6\times {\left(\frac{t}{T}\right)}^{5}]\\ y\left(t\right)=y_{0}+(y_{f}-y_{0})\times [10\times {\left(\frac{t}{T}\right)}^{3}-15\times {\left(\frac{t}{T}\right)}^{4}+6\times {\left(\frac{t}{T}\right)}^{5}] \end{array}$$

where *x*_0_ and *x*_*f*_ are the initial and final positions, respectively, *T* is the duration of the reaching movement and *t* is a vector representing time. Hand velocity was calculated from the derivative of the position with respect to time and hand acceleration from the derivative of the velocity with respect to time. Hand movement characteristics were determined from previous studies on reaching. The reaching amplitude was 0.4 m at the resultant peak hand velocity of 1.73 m/s with the movement lasting 0.4 s. The target to be reached was positioned straight-ahead with respect to the body midline (see Figure 1 – lower panels). Because the simulated reaching movement was in the horizontal plane, Coriolis force deviated the hand only along the horizontal plane, therefore only joint torques around the vertical axis could correct hand deviations. Accordingly, torque due to gravity was not considered. As well, viscosity and elasticity due to tendons and muscles were not included in the equations of motion.

First, elbow (*θ*_1_) and shoulder (*θ*_2_) angles were determined from the right hand trajectory (see Figure 1 – upper panels) using inverse kinematics equations (Equations 2 and 3) for a two-link model:

(2) $$\theta_{2}=\cos^{-1}\left(\frac{x^{2}+y^{2}-L_{1}^{2}-L_{2}^{2}}{2\times L_{1}\times L_{2}}\right)$$

(3) $$\theta_{1}=\tan^{-1}\left(\frac{y}{x}\right)-\tan^{-1}\left(\frac{L_{2}\times \sin\left(\theta_{2}\right)}{L_{1}+L_{2}\times \cos\left(\theta_{2}\right)}\right)$$

where *L*_1_ and *L*_2_ are the length (m) of the upper and lower arm segments, respectively, and *x* and *y* are the coordinates of the hand position in a shoulder-centered system.

**Figure 2:**
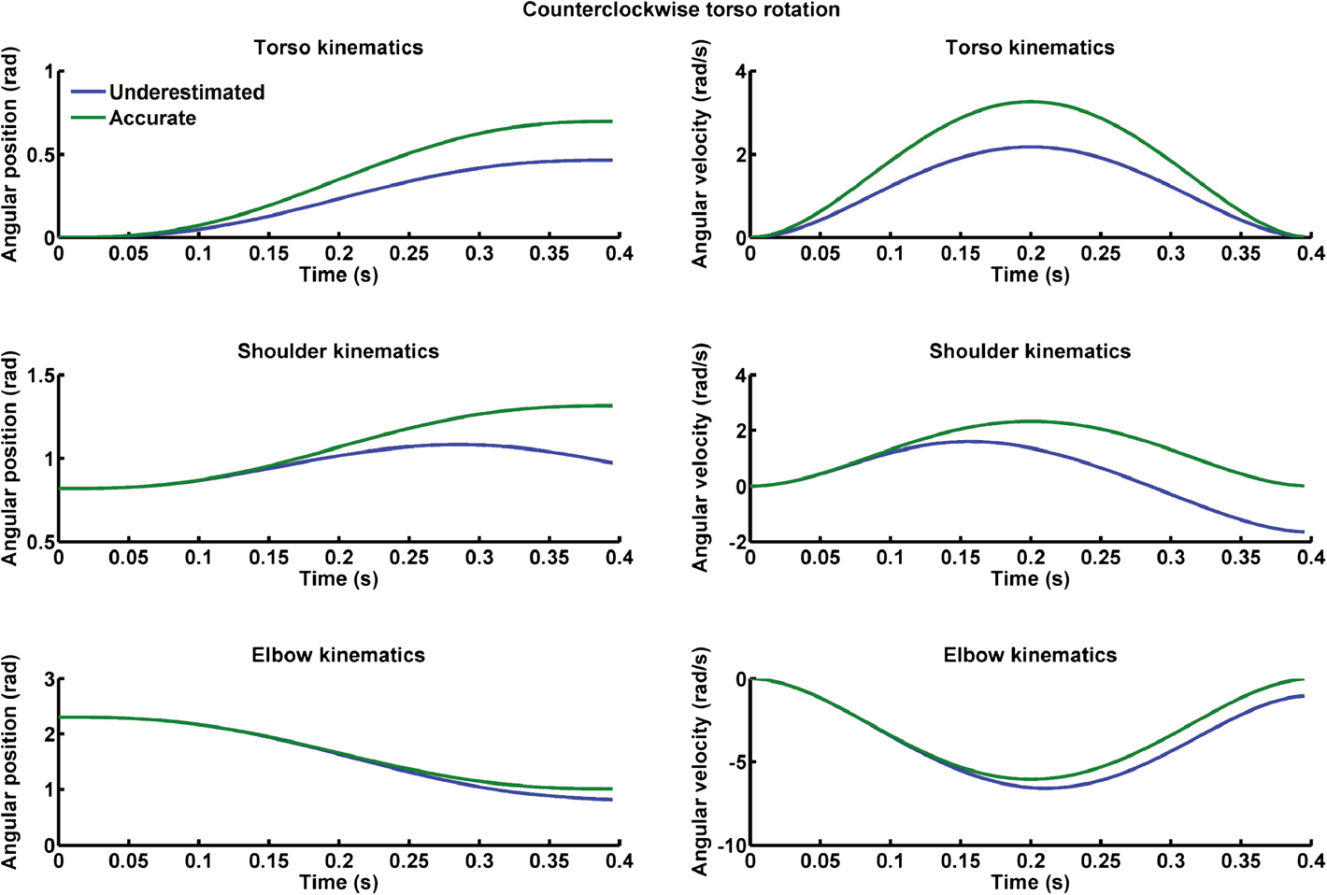
**Left column) Angular position for accurate (green lines) and underestimated (blue lines) counter clockwise torso rotations.** Right column) Angular velocity for accurate (green lines) and underestimated (blue lines) counter clockwise torso rotations. Upper panels depict torso kinematics, middle panels shoulder kinematics and lower panels elbow kinematics.

**Figure 3:**
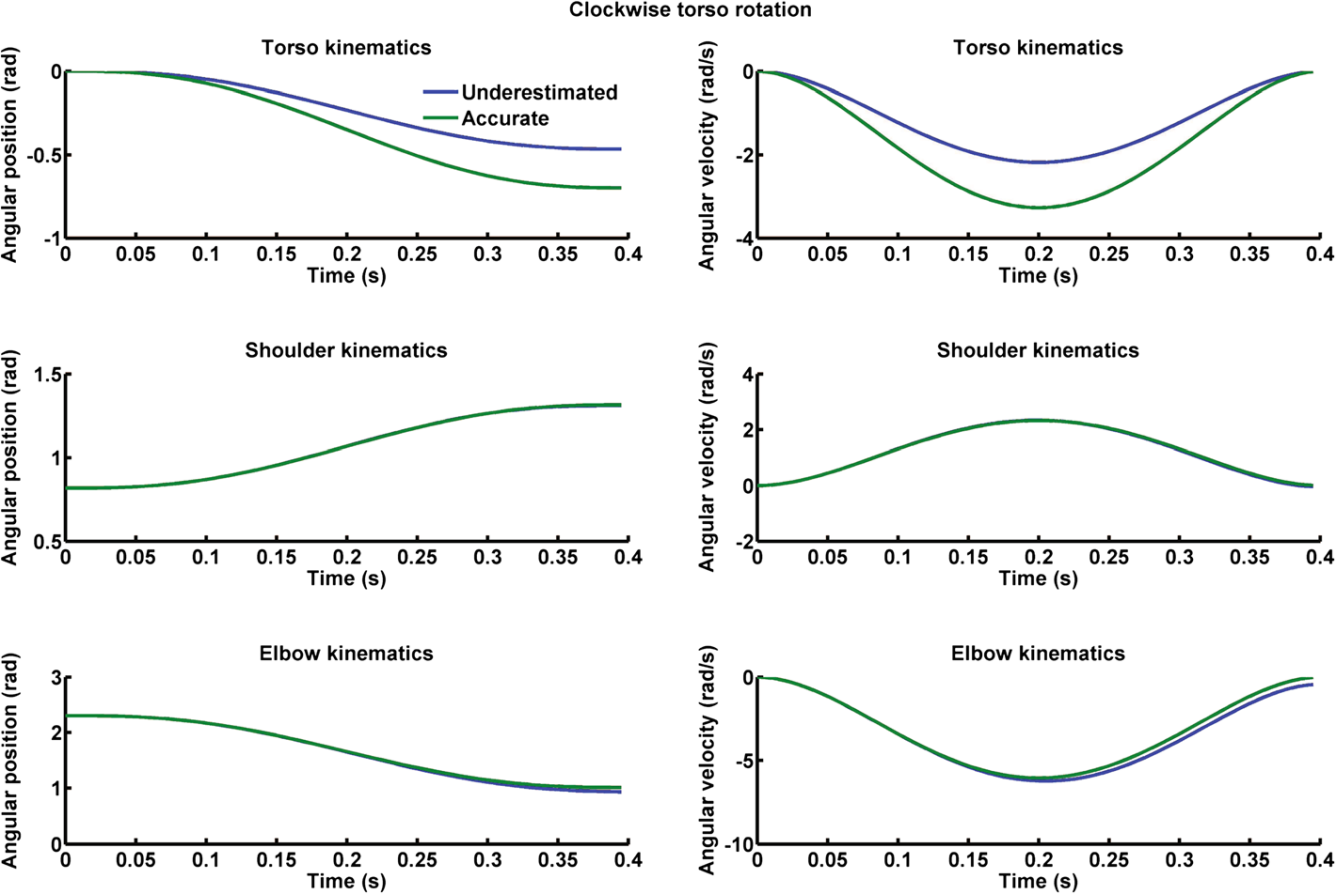
**Left column) Angular position for accurate (green lines) and underestimated (blue lines) clockwise torso rotations.** Right column) Angular velocity for accurate (green lines) and underestimated (blue lines) clockwise torso rotations. Upper panels depict torso kinematics, middle panels shoulder kinematics and lower panels elbow kinematics.

Thereafter, following Pigeon et al. [5], the torques $\vec{\tau }={\left(\tau_{1},\tau_{2},\tau_{3}\right)}^{T}$ at the shoulder, elbow and trunk joints were calculated from joint angles $\vec{\theta }={\left(\theta_{1},\theta_{2},\theta_{3}\right)}^{T}$ using inverse dynamics (equation 4). Trunk rotation and shoulder and elbow flexion in the counter clockwise direction were all considered positive. For the elbow and shoulder joints, an angular position of 0° occurred when the upper arm was collinear with both shoulders and when the elbow was fully extended (i.e., upper and lower arms parallel to the x axis: Figure 1 – lower right panel). Shoulder and elbow joint angular velocities and acceleration components were obtained by numerical differentiation. Torso rotation around the vertical axis is *θ*_3_. Counter clockwise and clockwise torso kinematics were simulated using an adapted version (i.e., Cartesian initial and final position were replaced by initial and angular position) of Equation 1. To simulate underestimation of torso rotation kinematics, we multiplied the accurate torso kinematics time-series by 1/G where G was a scalar ranging from 1.5 to 1. This procedure permitted us to scale down the accurate kinematics of torso rotation to create the underestimated kinematics (see Figures 2 and 3). To assess the magnitude of the underestimation in torso rotation kinematics, we determined the maximum trunk accelerations of the accurate and each of the underestimated torso kinematics time-series. Thereafter, we performed a rule of three: underestimation of trunk kinematics (%) = 100 - ((underestimated peak trunk acceleration × 100)/accurate peak trunk acceleration). Underestimations of trunk rotations were simulated from 0% (i.e., no torso kinematic error: accurate lines in Figures 2 and 3) to 33% (larger error in perception of torso rotation: underestimated lines in Figures 2 and 3).

(4) $$\begin{array}{l} {\vec{\tau }}_{\mathrm{net}}={\vec{\tau }}_{\mathrm{Normal}\;\mathrm{inertial}}+{\vec{\tau }}_{\mathrm{inertial}\mathrm{int}\mathrm{eraction}}\;+{\vec{\tau }}_{\mathrm{Centripetal}}\;+{\vec{\tau }}_{\mathrm{Coriolis}}\\ {\vec{\tau }}_{1}\;=H_{4}{\ddot{\theta }}_{4}\;+\left(H_{2}{\ddot{\theta }}_{3}+H_{5}{\ddot{\theta }}_{2}\right)+(h_{3}{\dot{\theta }}_{3}^{2}+h_{4}{\dot{\theta }}_{2}^{2})\\ \;+(2h_{4}{\dot{\theta }}_{3}{\dot{\theta }}_{2}+2h_{4}{\dot{\theta }}_{1}{\dot{\theta }}_{2})\\ {\vec{\tau }}_{2}=H_{6}{\ddot{\theta }}_{2}\;+\left(H_{3}{\ddot{\theta }}_{3}+H_{5}{\ddot{\theta }}_{1}\right)+(h_{5}{\dot{\theta }}_{3}^{2}+h_{6}{\dot{\theta }}_{1}^{2})\\ \;+(2h_{6}{\dot{\theta }}_{3}{\dot{\theta }}_{1})\;\\ {\vec{\tau }}_{3}=H_{1}{\ddot{\theta }}_{3}\;+\left(H_{2}{\ddot{\theta }}_{1}+H_{3}{\ddot{\theta }}_{2}\right)+(h_{1}{\dot{\theta }}_{1}^{2}+h_{2}{\dot{\theta }}_{2}^{2})\\ \;+(2h_{1}{\dot{\theta }}_{3}{\dot{\theta }}_{1}+2h_{2}{\dot{\theta }}_{3}{\dot{\theta }}_{2}+2h_{2}{\dot{\theta }}_{1}{\dot{\theta }}_{2}) \end{array}$$

Equation 5 is a simplification of Equation 4.

(5) $$\vec{\tau }=H\vec{\ddot{\theta }}+\vec{A}$$

In Equation 5, H and $\vec{A}$ are:

$$\begin{array}{l} H=\left(\begin{array}{ccc} H_{4} & H_{5} & H_{2}\\ H_{5} & H_{6} & H_{3}\\ H_{2} & H_{3} & H_{1} \end{array}\right)\\ \vec{A}=\left(\begin{array}{c} h_{3}{\dot{\theta }}_{3}^{2}+h_{4}{\dot{\theta }}_{2}^{2}+2h_{4}{\dot{\theta }}_{3}{\dot{\theta }}_{2}+2h_{4}{\dot{\theta }}_{1}{\dot{\theta }}_{1}\\ h_{5}{\dot{\theta }}_{3}^{2}+h_{6}{\dot{\theta }}_{1}^{2}+2h_{6}{\dot{\theta }}_{3}{\dot{\theta }}_{1}\\ h_{1}{\dot{\theta }}_{1}^{2}+h_{2}{\dot{\theta }}_{2}^{2}+2h_{1}{\dot{\theta }}_{3}{\dot{\theta }}_{1}+2h_{2}{\dot{\theta }}_{3}{\dot{\theta }}_{2}+2h_{2}{\dot{\theta }}_{1}{\dot{\theta }}_{2} \end{array}\right) \end{array}$$

Matrix H and $\vec{A}$ are described in detail by Pigeon et al., 2003. The anthropometric data were related to the height (1.78 m) and mass (80 kg) of the model according to the literature. Segment length (L), expressed as a percentage of body height, was drawn from [27]. Segment mass (m), moment of inertia (I) and distance to the centre of mass from the proximal joint (r) were computed from Dempster’s table [28].

To predict joint angles from the torques (i.e., forward dynamics), Equation 5 was inverted.

(6) $$\vec{\ddot{\theta }}=H^{-1}\left(\vec{\tau }-\vec{A}\right)$$

Shoulder and elbow joint torques (i.e., Coriolis, centripetal, inertial interaction and normal inertial torques) were calculated (i.e., inverse dynamics, Equation 7) in relation to the underestimation of trunk rotation kinematics (the hats over ${\hat{\ddot{\theta }}}_{3}$ and ${\hat{\dot{\theta }}}_{3}$ indicate that the underestimated torso kinematics were used to calculate either joint torques (Equation 7) or angular kinematics (Equation 8)):

$$\begin{array}{l} \hat{\ddot{\theta }}=\left[\begin{array}{c} {\ddot{\theta }}_{1}\\ {\ddot{\theta }}_{2}\\ {\hat{\ddot{\theta }}}_{3} \end{array}\right]\;\mathrm{and}\;\mathrm{in}\\ \hat{\vec{A}}=\left(\begin{array}{c} h_{3}{\hat{\dot{\theta }}}_{3}^{2}+h_{4}{\dot{\theta }}_{2}^{2}+2h_{4}{\hat{\dot{\theta }}}_{3}{\dot{\theta }}_{2}+2h_{4}{\dot{\theta }}_{1}{\dot{\theta }}_{1}\\ h_{5}{\hat{\dot{\theta }}}_{3}^{2}+h_{6}{\dot{\theta }}_{1}^{2}+2h_{6}{\hat{\dot{\theta }}}_{3}{\dot{\theta }}_{1}\\ h_{1}{\dot{\theta }}_{1}^{2}+h_{2}{\dot{\theta }}_{2}^{2}+2h_{1}{\hat{\dot{\theta }}}_{3}{\dot{\theta }}_{1}+2h_{2}{\hat{\dot{\theta }}}_{3}{\dot{\theta }}_{2}+2h_{2}{\dot{\theta }}_{1}{\dot{\theta }}_{2} \end{array}\right) \end{array}$$

(7) $$\hat{\vec{\tau }}=H\hat{\vec{\ddot{\theta }}}+\hat{\vec{A}}$$

From Equation 7, the predicted shoulder and elbow joint torques were altered due to the underestimation of torso rotation. Thereafter, these altered joint torques ( $\hat{\vec{\tau }}$) were used in equation 8 as input into the forward dynamic equation to determine elbow and shoulder joint kinematics:

(8) $$\vec{\ddot{\theta }}=H^{-1}\left(\hat{\vec{\tau }}-\vec{A}\right)$$

Then, from the elbow and shoulder joint angular kinematics, hand trajectories were determined using forward kinematics.

## Results

**Figure 4:**
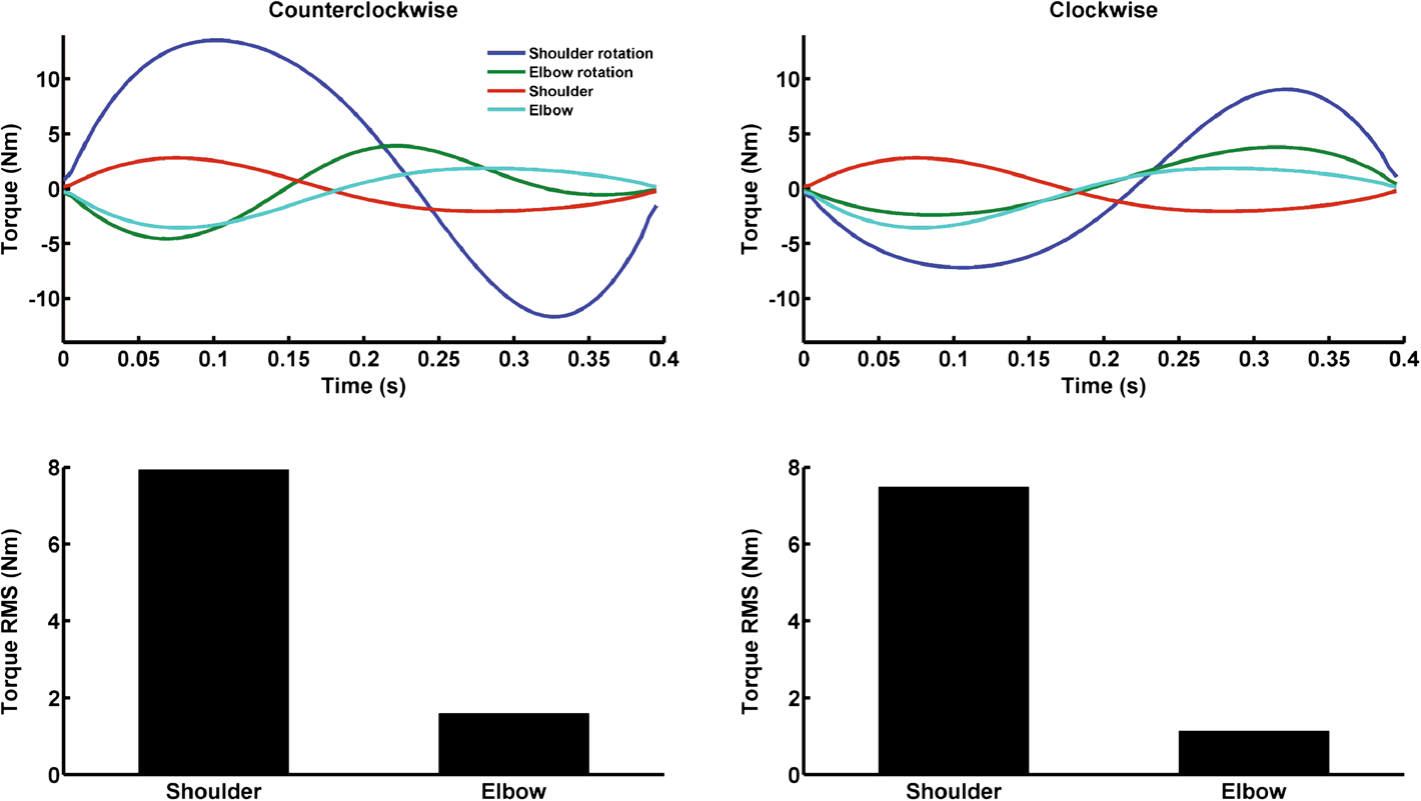
**Net shoulder and elbow joint torques required to reach the target in the absence of torso rotation (Shoulder and Elbow lines) and during counter clockwise (left upper panel) or clockwise rotations (right upper panel).** Root mean square (RMS) of net shoulder and elbow joint torques for counter clockwise (left lower panel) and clockwise (right lower panel) rotations.

Although it was expected that the net shoulder and elbow joint torques would be altered when reaching during torso rotation (to avoid hand trajectory deviation and ensure accurate reaching), it remained to be determined how the net joint torques would be altered and whether these changes would depend on the direction of torso rotation. To our knowledge, no study has calculated the change in net shoulder and elbow joint torques that allow for accurate reaching movements during counter clockwise and clockwise torso rotations. The outcomes of the biomechanical model revealed that regardless of the direction of torso rotation, the increase in net shoulder joint torque was much larger than the increase in net elbow joint torque (Figure 4 - upper panels). For instance, the mean changes in net torques (i.e., root mean square value of the difference between the torque during torso rotation compared to the torque in the absence of torso rotation: Figure 4 – lower panels) during counter clockwise rotation were 7.93 Nm and 1.59 Nm the for shoulder and elbow joints, respectively. During clockwise rotation, the computed torque changes were 7.48 Nm (shoulder) and 1.13 Nm (elbow). Overall, the increase in both net torques secondary to torso rotation was similar and did not depend upon the direction of the rotation.

**Figure 5:**
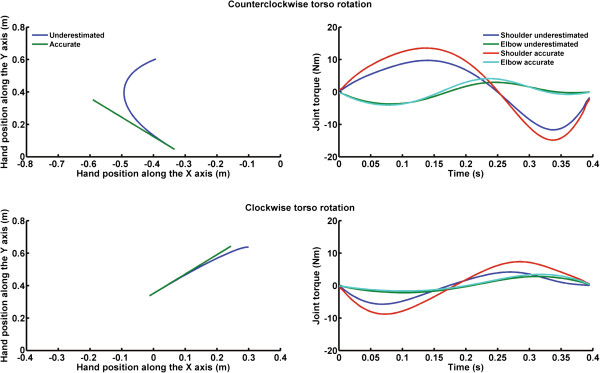
**Upper left panel) Hand trajectories for accurate estimation of counter clockwise torso rotation (green lines) and for the larger underestimation of counter clockwise torso rotation (blue line).** Upper right panel) Net shoulder and elbow joint torques (i.e., shoulder accurate and elbow accurate lines) during accurate perception of torso rotation, resulting in accurate hand trajectory, and net shoulder and elbow joint torques (i.e., shoulder underestimation and elbow underestimation lines) during the larger underestimation of counter clockwise torso rotation, resulting in underestimated hand trajectory. Lower panels) Same caption, but data are for clockwise torso rotation.

When underestimating the acceleration of torso rotation (i.e., larger torso acceleration underestimation: 33%), the biomechanical model showed that the hand deviated from a straight-ahead trajectory in the opposite direction of the rotation (i.e., in the direction of the Coriolis force generated on the arm: Figure 5 upper and lower left panels). Remarkably, the hand trajectory deviated further during counter clockwise as opposed to clockwise torso rotation. This was irrespective of the fact that the changes in net shoulder and elbow joint torque due to the underestimation of torso rotation were similar (RMS value calculated between joint torques accurate and underestimated torso rotation: shoulder = 2.53 Nm and 2.77 Nm and elbow joint = 0.46 Nm and 0.66 Nm, for clockwise and counter clockwise rotation, respectively).

**Figure 6:**
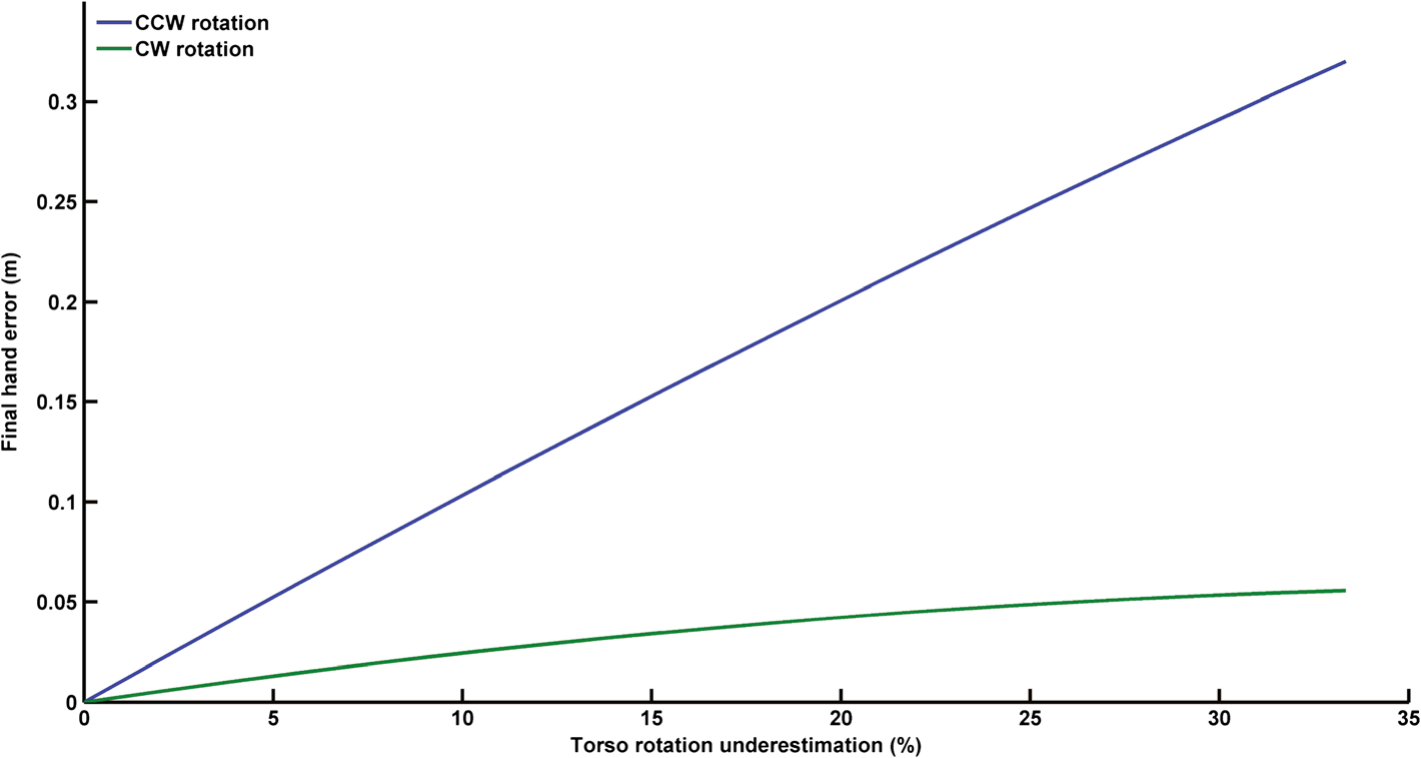
Final hand error as a function of the percentage of the underestimation of counter clockwise (blue line) and clockwise torso rotation (green line).

We computed the final hand error by measuring the Euclidean distance, at movement offset, between the hand position measured when torso rotation was underestimated and when it was correctly perceived. The errors increased along with the magnitude by which the torso rotation was underestimated (Figure 6). It is worth noting that the effect of misjudging trunk rotation on final hand error was greater for counter clockwise than for clockwise rotations. For instance, underestimating counter clockwise torso rotation by only 10% induced a final hand deviation as large as 11.1 cm. Comparatively, as the underestimation of trunk rotation in the clockwise direction increased, the final hand angular error did not increase considerably. For example, underestimating the trunk rotation by 33% led to a final hand deviation of only 3.8 cm. In contrast, the same degree of perceptual error for counter clockwise rotation resulted in a final hand deviation of 32.2 cm (i.e., ~8.5 times larger).

**Figure 7:**
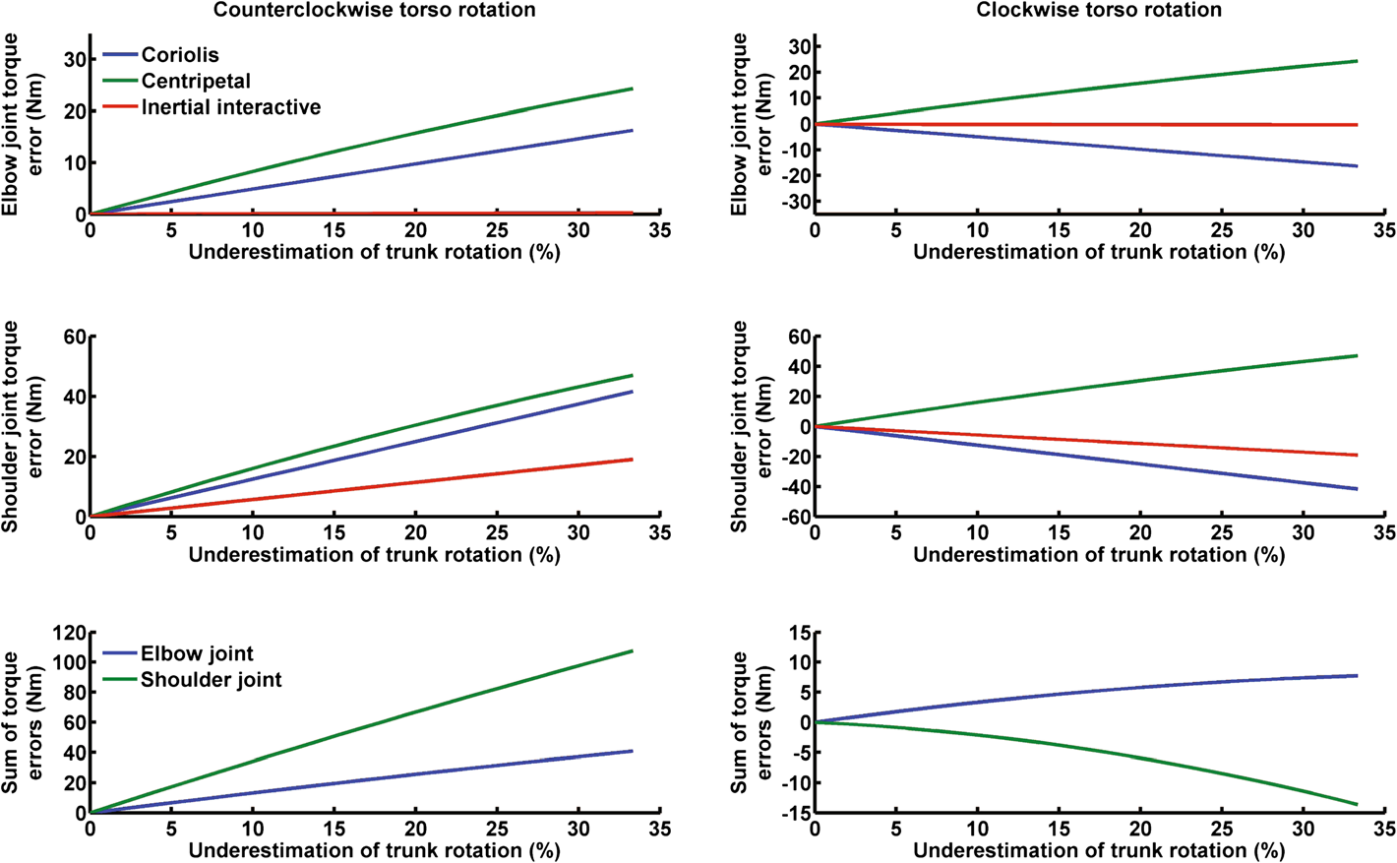
**Left upper and middle panels) Coriolis, centripetal and inertial interactive torque errors for the elbow and shoulder joints as a function of the percentage of the underestimation of counter clockwise torso rotation.** Lower left panel) Sum of torque errors for the elbow and shoulder joints as a function of the percentage of the underestimation of counter clockwise torso rotation. Right upper and middle panels) Coriolis, centripetal and inertial interactive torque error for elbow and shoulder joints as a function of the percentage of the underestimation of clockwise torso rotation. Lower right panel) Sum of torque errors for the elbow and shoulder joints as a function of the percentage of the underestimation of clockwise torso rotation.

To gain insight into the differences in final hand error between rotational directions, we calculated the difference in the torque time-series between accurate (i.e., torso rotation underestimation = 0%) and underestimated trunk rotations (i.e., torso rotation underestimation > 0%) for the Coriolis, centripetal, inertial interactive and normal interactive torques (note that the normal interactive torque was unaffected by torso rotation therefore it is not illustrated in Figure 7). Thereafter, we summed the difference. This calculation was repeated for every percentage of torso rotation underestimation. The result of this calculation was called torque error (elbow and shoulder joint torque errors: Figure 7 – upper and middle panels). For each joint, we also summed the Coriolis, centripetal and inertial interactive torque errors (sum of torque errors: Figure 7 - lower panels). A joint torque error larger than zero indicates that the joint torque during underestimated torso rotation was smaller than the torque for accurate perception of torso rotation (i.e., torques leading to accurate straight-ahead reaching trajectory despite trunk rotation). In contrast, a joint torque error smaller than zero implies that the joint torque generated during underestimated torso rotation was larger than the torque for accurate perception of torso rotation.

**Figure 8:**
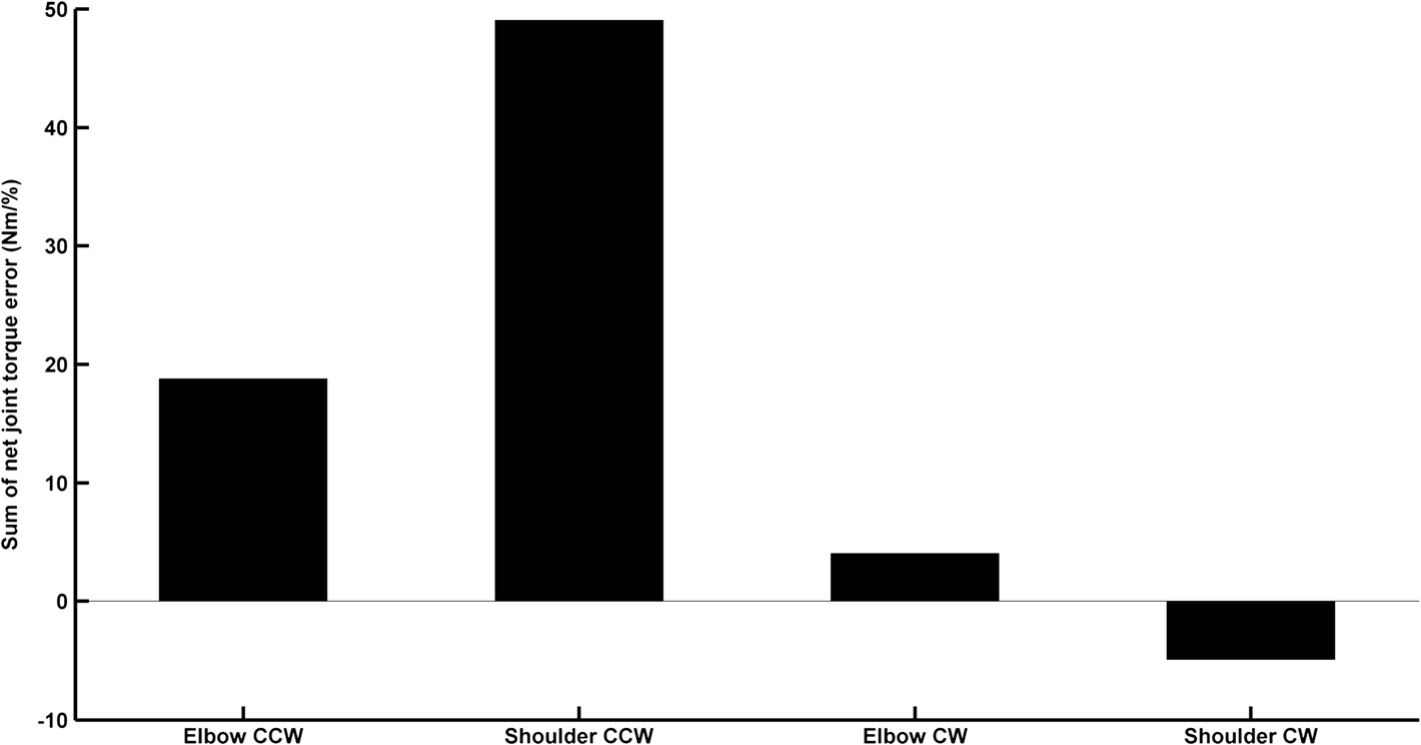
Sum of net elbow and net shoulder joints torque errors per percentage of torso rotation underestimation for counter clockwise (CCW) and clockwise (CW) rotation.

Because the underestimation of clockwise rotations led to smaller hand deviations than counter clockwise rotations, joint torque errors may have been a function of trunk direction. Consequently, it is expected that the elbow and shoulder joint torque error should approach zero during clockwise rotation. The analysis of each torque error revealed that at the elbow joint, contrary to counter clockwise rotations, the Coriolis torque was in the opposite direction of the centripetal torque during clockwise rotations (Figure 7: upper right panel vs. upper left panel). Therefore, during clockwise rotation, these torques acted primarily to counterbalance each other. This is evidenced by the sum of elbow joint errors for clockwise rotation (Figure 7: lower right panel), which is close to zero. Furthermore, similar patterns are observed for the Coriolis and centripetal shoulder joint torques. Contrary to counter clockwise rotation, these torques cancelled each other during clockwise rotation (Figure 7: middle right panel). For counter clockwise rotations Coriolis and centripetal torques evolved in the same direction for both joints (Figure 7: upper and middle left panels). As a result, the sum of the torque errors at both joints was larger for counter clockwise versus clockwise rotation (Figure 7: lower left panel). Consequently, the torques pushed the arm to a greater extent during counter clockwise rotations, leading to greater deviations in trajectory and final hand error in the former condition. It is worth mentioning that regardless of rotational direction, underestimating trunk rotation had a negligible effect on the elbow joint inertial interaction torque error. However, it did result in a marginal increase at the shoulder joint in both directions. Finally, the sum of both net joint errors, normalized by the percentage of underestimation, demonstrated that the net elbow and shoulder joints torque errors are approximately 5 and 12 times larger for counter clockwise compared to clockwise rotation (Figure 8).

## Discussion

Torso rotation while reaching for an object creates additional torques (e.g., Coriolis) on the arm that must be accounted for by motor commands in order to ensure accurate movement. As the magnitudes of these torques depend on trunk angular kinematics, misperception of trunk rotation may therefore alter reaching accuracy. The feedforward 2-D biomechanical model presented herein aimed to assess the consequences of underestimating torso rotation on reaching accuracy and to determine whether these consequences depend on the direction of torso rotation.

The outcomes of the current model support the model proposed by Bortolami et al. [11]. Their model showed that for counter clockwise rotations, in comparison to reaching in the absence of torso rotation, the amplitudes of the net shoulder and elbow torques are scaled up to ensure accurate final hand position despite body rotations. However, this model was not developed to determine the consequences of the direction and the misperception of torso rotations on reaching accuracy. The novel observation provided by the present model is that the accuracy of reaching movements performed during trunk rotation decreases with increasing underestimations of trunk rotation. As well, this model shows that the amplitude of the final hand error is larger for counter clockwise rotations than for clockwise rotations. Indeed, regardless of the magnitude of the underestimation for clockwise rotation, the Coriolis and centripetal torques partly cancel out, thereby reducing the detrimental effect of errors in perceiving trunk rotation on right hand trajectory. As these torques acted in the same direction for counter clockwise trunk rotations, the resulting net sum of residual torques at the shoulder and elbow joints were considerable. In this case, movement accuracy was largely affected by rotational underestimations. If the simulated reaching movement had been performed with the left hand, the underestimation of clockwise rotation would have induced a larger final hand error as the Coriolis and Centripetal torque errors would not, in this case, cancel each other out. In contrast, a smaller final hand error would be observed for left hand reaching during counter clockwise rotation.

Our feedforward model simulated error (i.e., underestimation of torso acceleration) occurring during the planning stage of reaching. Therefore, it excluded any online correction of hand deviation based on arm proprioception or vestibular and visual information (i.e., feedback control). According to current motor control models, for self-generated torso and reaching movements, the brain may use motor commands in conjunction with internal models of both the arm and trunk to anticipate the resultant perturbing torques and thereby adjust the arm motor commands in a feedforward manner e.g., [29]. For example, while reaching in the absence of trunk movement, muscle activity in the shoulder and elbow joints varies in a predictive manner to compensate for interaction torques arising from multi-joint dynamics [3,30-32]. Therefore, to reach accurately while the torso is rotating, the brain likely uses internal models to predict and offset the kinematic consequences of intersegmental dynamics [5,16,17]. Sensory information is crucial to develop, maintain and update such internal models e.g., [33,34]. Consequently, accurate internal models of the trunk and arm are essential to perform accurate reaching movements during voluntary head and torso rotations. Based on this proposition and our model, hand movement inaccuracies observed in patients with vestibular defects most likely result from an underestimation of the mechanical consequences of trunk movements on their arm [35-37]. In addition, patients with moderate and severe impairments in a paretic arm will move their trunk to reach an object even if it is not necessary [4]. Therefore, it is possible that any reaching inaccuracy they experience may, in part, be due to an imprecise internal model of trunk motion. Consequently, training programs aiming to improve reaching movements in these populations should involve exercises implying arm movements towards various target locations during trunk rotations. Furthermore, the present results suggest that when designing upper limb prostheses controllers, adding a sensor that monitors trunk kinematics could improve prostheses control and performance as torso motions would be taken into account by the controller.

### Potential limitations

Our feedforward model did not attempt to evaluate the use of sensory cues related to trunk kinematics to correct hand trajectory during the movement (i.e., online correction based on error-feedback signals). It is likely that these cues (e.g. vestibular), in conjunction with the monitoring of the motor commands, offer enough information to control self-induced interaction torques as they arise during torso rotation. Nonetheless, well-learned movements such as manual reaching do not heavily rely on continuous feedback control. For example, adaptation studies have revealed that a sudden change in the inertial configuration of the arm during reaching induces initial errors in reaching that can be well predicted by an open-looped forward model [38]. On the other hand, the lack of experimental data to validate the outcomes of the biomechanical model could be seen as another limitation of the present study. The acquisition of such data would require us to determine the perception of torso rotation, either in healthy individuals or subjects with neurological pathologies. While this can be performed relatively easily after the rotations [39-41], assessing real-time errors in the perception of torso rotation kinematics proves to be much more challenging, especially when subjects are involved in a concomitant reaching task. Finally, to further explore the effect of torso rotation misperception on reaching accuracy, it would be informative to assess the effect of arm movements in different directions relative to the body and to gravity.

## Conclusion

The present study demonstrates that even small errors in perceiving or predicting the kinematics of counter clockwise torso rotation may impair the accuracy of reaching movements. However, errors in estimating clockwise rotation appear less detrimental to movement accuracy as in this instance, the shoulder and elbow joint torques work to effectively reduce hand deviation. Finally, according to the outcomes of the feed forward model, healthy individuals likely possess accurate internal models of their arm and torso kinematics so that they normally show small errors when reaching for a target while simultaneously rotating their trunk [5,42].

## Endnote

^1^As vestibular receptors respond to head acceleration, the perception of body motion is impaired when the body rotates at constant velocity. The large reaching errors that have been reported during such rotations [6-9] suggest that proprioceptive and cutaneous inputs provide little information about body rotations.

## Competing interests

There are no conflicts of interest to report. The authors have not received any payment for conducting this work.

## Authors’ contributions

MS developed the biomechanical model. Each author participated in the drafting of the manuscript. All authors approved the final manuscript.
